# Prognostic value of lymphocyte-to-monocyte ratio in ovarian cancer: a meta-analysis

**DOI:** 10.1186/s13048-019-0527-z

**Published:** 2019-05-31

**Authors:** Jun Gong, Hui Jiang, Chang Shu, Mei-qin Hu, Yan Huang, Qin Liu, Rong-feng Li

**Affiliations:** 1 Department of Abdominal and Pelvic Medical Oncology, Huangshi Central Hospital, Affiliated Hospital of Hubei Polytechnic University, Edong Healthcare Group, Hubei Province Huangshi, People’s Republic of China; 2 Department of Urology, Huangshi Central Hospital of Edong Healthcare Group, Hubei Polytechnic University, Huangshi, Hubei China; 3 Department of Clinical Laboratory, Huangshi Central Hospital of Edong Healthcare Group, Hubei Polytechnic University, Huangshi, Hubei China; 4 Department of Breast surgery, Thyroid surgery, Huangshi Central Hospital of Edong Healthcare Group, Hubei Polytechnic University, Huangshi, Hubei China; 5 Department of Abdominal and Pelvic Medical Oncology, Huangshi Central Hospital, Hubei Polytechnic University, No.141, Tianjin Road, Huangshi, 435000 Hubei China

**Keywords:** Lymphocyte-to-monocyte ratio, Meta-analysis, Ovarian cancer, Prognosis

## Abstract

**Introduction:**

Prognostic biomarkers are highly needed to properly manage patients with cancer and improve their clinical courses. The relationship between lymphocyte-to-monocyte ratio (LMR) at diagnosis and ovarian cancer prognosis has been extensively studied, but little consensus has been reached regarding its utility as a biomarker of poor outcome. Thus, this study aimed to investigate the potential prognostic value of pretreatment LMR in such patients to shed light on this issue.

**Methods:**

We searched the scientific databases of MEDLINE, Embase, Cochrane Library, and WangFang for relevant studies about the inflammatory prognostic factor LMR in ovarian cancer, based on specific inclusion and exclusion criteria. The following parameters were analyzed among others: LMR values and respective cut-offs, patient’s overall survival (OS) and progression-free survival (PFS), and clinicopathological features.

**Results:**

Eight studies, including 2259 patients, were eligible for inclusion in this meta-analysis. We found that low LMR was associated with both poor OS [Hazard ratio (HR): 1.92; 95% confidence interval (CI): 1.58–2.34; *p* < 0.001] and PFS (HR: 1.70; 95% CI: 1.54–1.88; p < 0.001). Moreover, our findings revealed that low LMR was correlated with high G2/G3 histological grade (OR: 1.67; 95% CI: 1.26–2.20; p < 0.001) and late III-IV FIGO stage tumors (OR: 3.55; 95% CI: 2.68–4.70; p < 0.001), high serum CA-125 level (OR: 2.18; 95% CI: 1.71–2.77; p < 0.001), and presence of malignant ascites (OR: 1.87; 95% CI: 1.11–3.14; *p* = 0.02) and lymph node metastases (OR: 1.70; 95% CI: 1.13–2.54; *p* = 0.01).

**Conclusion:**

Pretreatment LMR is a potential prognostic marker of poor outcome in ovarian cancer patients and may thus be important in clinical care and disease control.

## Introduction

Ovarian cancer is one of the most common gynecological malignant tumors with the highest mortality rate. Over 90% of ovarian cancer is of epithelial origin, and non-epithelial tumors are usually derived from the granulosa or germ cells [1]. These differences in ovarian cancer etiology require different diagnostic approaches and result in distinct treatment regimens.

Despite advances in early diagnosis and targeted drug treatment, as well as improvements in drug cytotoxicity, most patients are still diagnosed at an advanced stage [2, 3]. Furthermore, chemotherapy is frequently not effective in controlling the disease mainly due to the development of primary or secondary resistance to anticancer drugs. In addition, some chemotherapy regimens are also associated with increased relapse and mortality rates among patients with ovarian cancer [4]. Therefore, better understanding of carcinogenic mechanisms is needed. The use of suitable and improved biomarkers could aid in both the diagnosis and prognosis of ovarian cancer.

It is well known that inflammatory and immune responses within the tumor microenvironment play important roles in tumorigenesis and cancer progression [5, 6]. Several inflammatory-related prognostic factors, such as the platelet-to-lymphocyte (PLR), neutrophil-to-lymphocyte (NLR), lymphocyte-to-monocyte (LMR), and C-reactive protein/albumin (CAR) ratios have been recently evaluated for their ability to predict outcomes of patients with various solid cancers [7–9]. In fact, PLR has been continuously reported as a novel inflammation-based prognostic index over the past years.

Similarly, LMR has been associated with a poor prognosis in several cancer types [10–12]. However, its prognostic value in ovarian cancer has not yet been fully elucidated. With this in mind, we decided to carry out this meta-analysis to elucidate the relationship of LMR with the clinicopathology of ovarian cancer and to establish whether it might be useful in predicting patient outcome.

## Materials and methods

### Search strategy

We searched the MEDLINE, Embase, Cochrane Library, and WanFang databases to identify the relevant articles using the search terms “LMR”, “lymphocyte to monocyte ratio”, “lymphocyte monocyte ratio”, or “lymphocyte-to-monocyte ratio” combined with “ovarian cancer”, “ovarian carcinoma”, “ovarian adenocarcinoma”, “ovarian tumor”, or “ovarian neoplasms”. The literature search was performed up to November 20, 2018.

### Inclusion and exclusion criteria

Published articles were selected for study based on the following inclusion criteria: (1) reported association between pretreatment LMR and prognosis in ovarian cancer; (2) patients grouped into “high LMR group” and “low LMR group” according to cut-off values of LMR; and (3) hazard ratios (HRs) with 95% confidence intervals (CIs) calculated for overall survival (OS), progression-free survival (PFS), or cancer-specific survival (CSS). The exclusion criteria were as follows: (1) lack of appropriate data; (2) duplicate publications; and (3) reviews, meta-analysis, letters, and conference abstracts.

### Data extraction and quality assessment

Data extraction was conducted independently by two investigators. The following information (on study details and clinopathological features) was collected from the studies: first author, year and country of study, number of patients involved and distribution of age and gender, tumor histological type, grade, stage, and optimal debulking, presence of malignant ascites and lymph node metastases, type of treatment applied (including surgery and chemotherapy dosage and duration), cut-off values of LMR, patient’s survival outcome (assessed by OS and PFS), and duration of follow-up period.

The Newcastle–Ottawa Scale (NOS) [13] was used to assess the methodological quality of the studies. According to this scale, the maximum score is 9; studies with NOS > 6 were considered high-quality studies.

### Statistical analysis

We used Review Manager 5.3 (Cochrane Collaboration, Oxford, UK) to pool HRs for OS and PFS and to pool odd ratios (ORs) for clinicopathological parameters. The HRs and 95% CIs were directly obtained from studies that included survival analysis or, when necessary, they were determined from the Kaplan-Meier curve by using Engauge Digitizer 4.1 [14, 15].

The heterogeneity across the eligible studies was assessed by the Cochran’s Q-test and I^2^ statistic. If I^2^ ≤ 50% or *p* > 0.05, indicating low heterogeneity, we used a fixed-effect model with an inverse variance method. Otherwise, we used a random-effect model with the DerSimonian and Laird method, which considers both within-and between-study variations [16]. A subgroup analysis was then performed to examine the potential source of heterogeneity. Sensitivity analysis was undertaken in order to test the robustness of the pooled results by removing each study. When more than eleven studies were included, Begg’s funnel plots and Egger’s linear regression tests were used to evaluate publication bias [17, 18]. In all analyses, a *p* value < 0.05 was considered statistically significant.

## Results

### Study selection

**Fig. 1:**
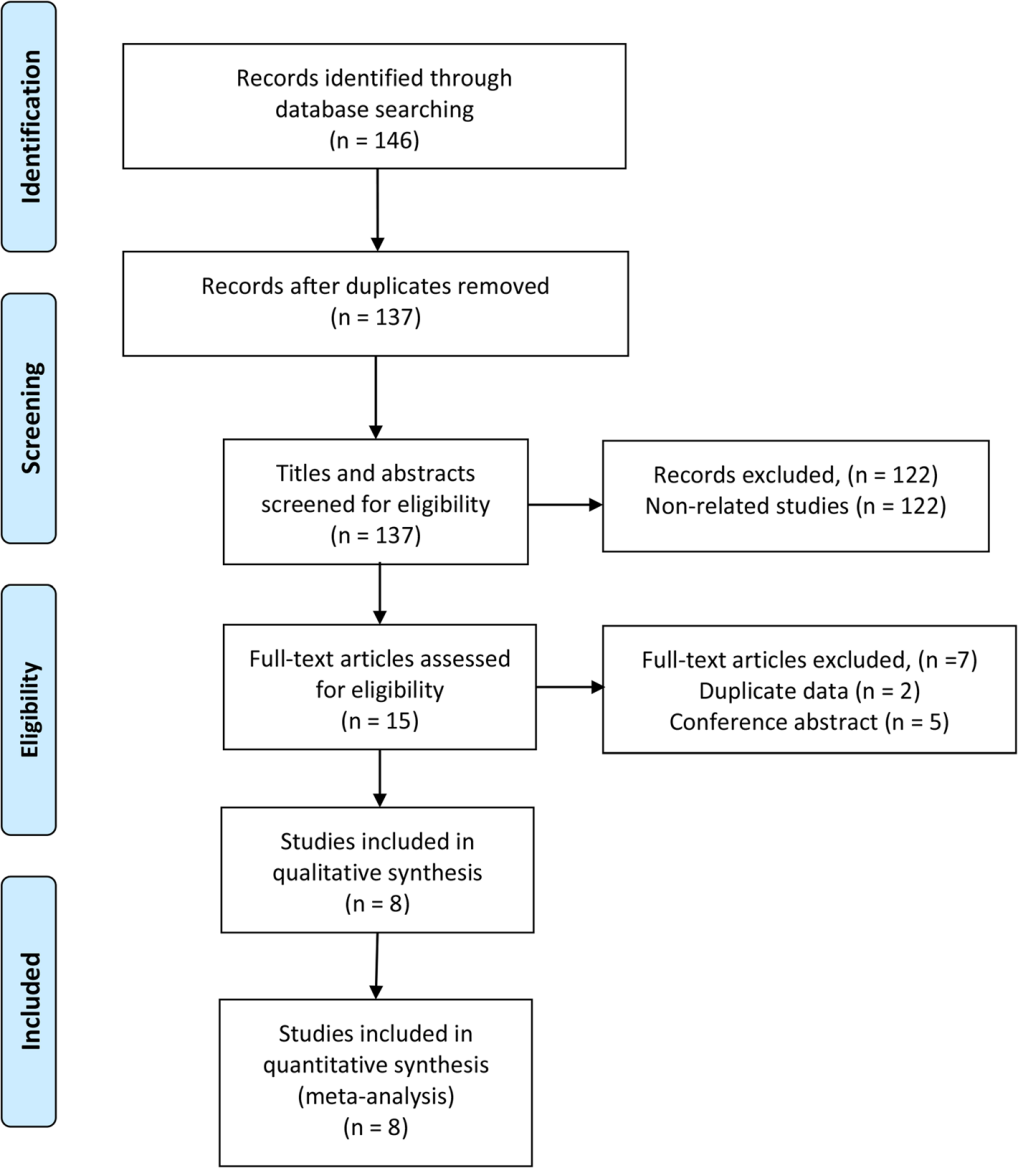
Flow diagram of study retrieval and selection processes

As shown in the flow diagram (Fig. 1), through the electronic search on the relevant databases, we initially retrieved 146 published articles, which were narrowed down to 137 following exclusion of duplicate studies and specific types of articles. After reviewing the title and abstract, 7 articles were excluded according to the exclusion criteria (i.e., lack of appropriate data) and 15 full-text articles were considered for further assessment. From these, 10 articles met the inclusion criteria and thus were used in the quantitative synthesis [19–25].

### Study characteristics

The main characteristics of the included studies are summarized in Table 1. They were all retrospective studies that were published between 2016 and 2018. There were six and two studies of mixed-stage (I–IV) and advanced-stage (III–IV) diseases, respectively, according to the International Federation of Gynaecology and Obstetrics (FIGO) criteria. All patients underwent surgery and adjuvant chemotherapy. All included studies assessed the prognostic value of LMR in OS, and only six in PFS. The cut-off values of LMR ranged from 1.85 to 4.2, which were determined in seven studies by the receiver operating curve sensitivity and specificity analysis (C-index); in one study, the method used was not reported [22]. Univariate and multivariate analysis were used to evaluate OS in one and seven studies, respectively. For all studies, the NOS scores were ≥ 6 (Table 1).

**Table 1:** Characteristics of the studies included in the meta-analysis

Author	Year	Country	Ethnicity	Follow-up (months)	Treatment	Age (years)	No. of patients	Stage	Cut-off value	Survival analysis	Analysis	NOS score
Temraz	2014	Lebanon	Caucasian	24	Mixed	65 (43–88)	68	Mixed	2.81	OS/RFS	UV	8
Lee	2015	UK	Caucasian	NA	Surgery	75 (65–81)	226	Early	1.8	OS	MV	7
Zhang	2015	China	Asian	50.8	Mixed	65 (30–78)	124	Mixed	4	OS	MV	8
Yoshida	2015	Japan	Asian	72 (27.6–111.6)	Mixed	72 (43–91)	181	Mixed	3.51	OS	MV	7
Lucca	2016	Austria	Caucasian	NA	Surgery	68 (61–74)	310	Early	3.3	OS	MV	6
D’Andrea	2017	Austria	Caucasian	42.4 (18.3–85.1)	Surgery	67 (60–73)	4198	Mixed	3.5	OS/RFS/CSS	MV	8
Miyake	2017	Japan	Asian	22 (10–64)	Mixed	72 (61–77)	117	Mixed	3.3	OS/CSS	UV	6
Rajwa	2018	Poland	Caucasian	14 (7–40)	Surgery	NA	144	Mixed	2.44	OS/CSS	MV	8
Wang	2018	China	Asian	NA	Mixed	63 (20–85)	270	Early	4	RFS	UV	7

Abbreviations: *OS* overall survival, *RFS* recurrence-free survival, *CSS* cancer-specific survival, *MV* multivariate, *NA* not available

### Meta-analysis

#### LMR and overall survival

**Fig. 2:**
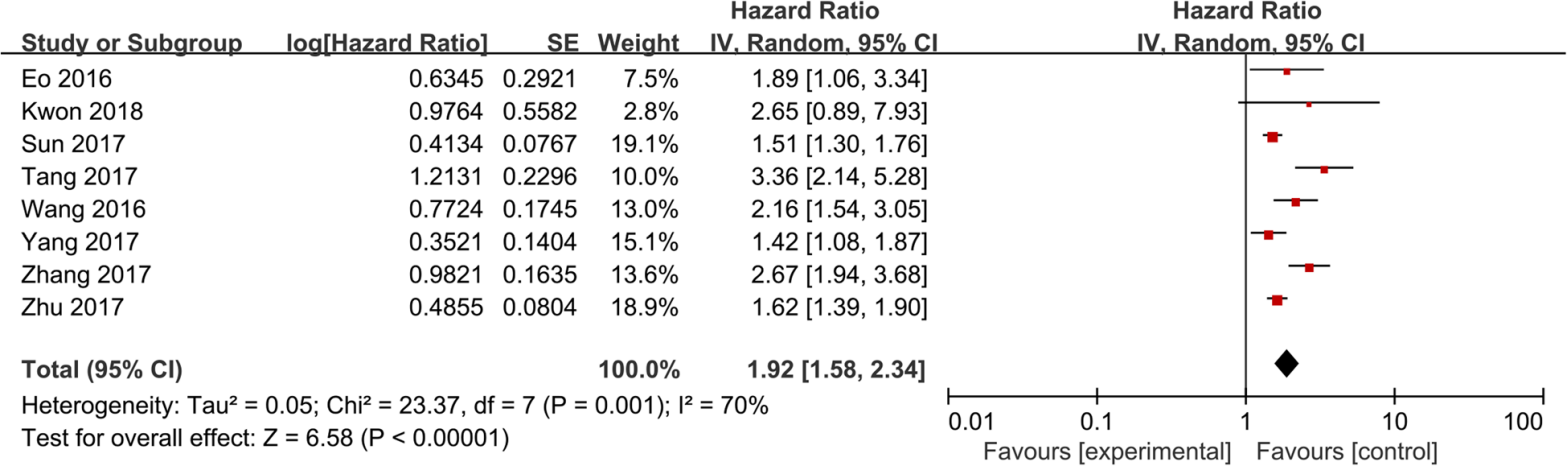
Pooled hazard ratio (HR) of lymphocyte-to-monocyte ratio (LMR) for overall survival (OS) in patients with ovarian cancer

Eight studies, comprising 2259 patients, investigated the predictive value of LMR in OS, revealing that a low LMR is indicative of a poor prognosis (worse OS) in ovarian cancer patients (HR: 1.92; 95% CI: 1.58–2.34; *p* < 0.001; Fig. 2). The test for high heterogeneity across the studies was significant (I^2^ = 70%; *p* = 0.001).

Subgroup analysis was then performed to further explore the prognostic value of LMR (Table 2). In agreement, the results showed that a low ratio significantly predicts a poor OS in patients with both mixed- (HR: 1.91; 95% CI: 1.47–2.47; p < 0.001) and advanced-stage disease (HR: 2.04; 95% CI: 1.26–3.31; p < 0.001). A similar relationship between LMR and OS was also detected in other subgroup analyses (*p* < 0.05).

**Table 2:** Pooled hazard ratios (HRs) for OS according to subgroup analyses

Subgroup	No. of studies	No. of patients	HR (95% CI)	P value	Heterogeneity	
					I^2^(%)	P_h_
Overall	8	5368	0.63 (0.50–0.80)	< 0.001	65.9	0.005
Ethnicity						
Asian	2	422	0.46 (0.25–0.87)	0.016	73.3	0.023
Caucasian	5	4946	0.80 (0.71–0.89)	< 0.001	8.2	0.360
Disease stage						
Early	2	536	0.61 (0.20–1.82)	0.377	0	0.705
Mixed	6	4832	0.63 (0.49–0.80)	< 0.001	75.3	0.001
Treatment						
Surgery	4	4878	0.81 (0.74–0.89)	< 0.001	0	0.883
Mixed	4	482	0.45 (0.27–0.73)	0.001	62.3	0.047
Cut-off for LMR						
≥ 3	5	4930	0.56 (0.35–0.88)	0.011	76.6	0.002
< 3	3	438	0.65 (0.41–1.04)	0.075	41.7	0.180
Analysis method						
Univariate	2	185	0.46 (0.27–0.79)	0.005	0	0.488
Multivariate	6	5183	0.67 (0.53–0.86)	0.001	65.9	0.005

#### LMR and progression-free survival

**Fig. 3:**
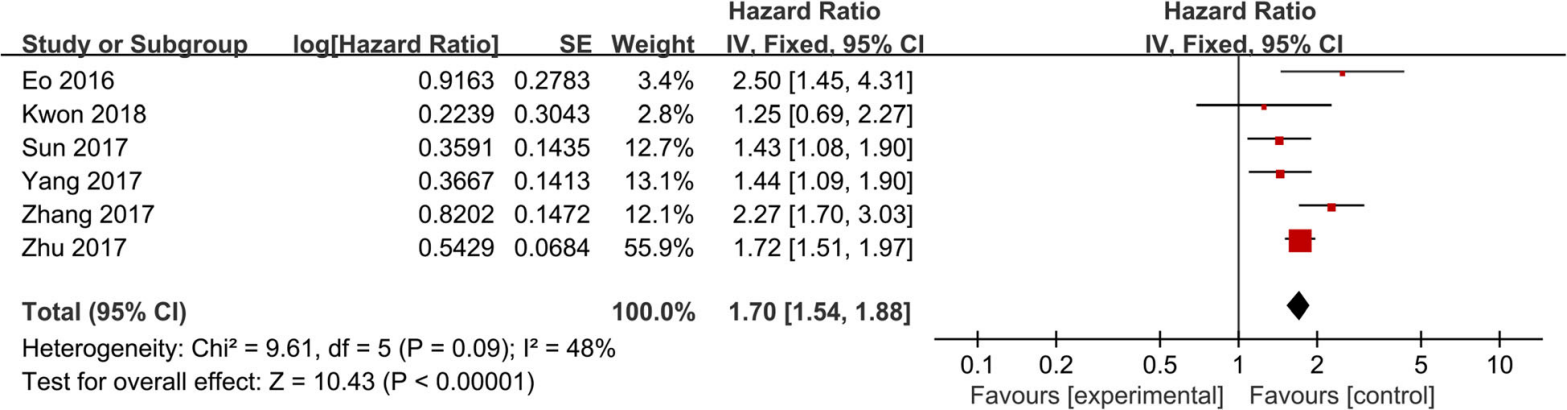
Pooled hazard ratio (HR) of lymphocyte-to-monocyte (LMR) for progression-free survival (PFS) in patients with ovarian cancer

Our findings showed a statistically significant negative relationship between LMR and PFS (Fig. 3), in which low values of LMR were associated with worse PFS (HR: 1.70; 95% CI: 1.54–1.88; *p* < 0.001). The results of the subgroup analyses based on FIGO stage, sample size, LMR cut-off value, and analysis method were similar to those of OS, meaning that in all cases a low ratio was also predictive of a poor PFS.

#### LMR and clinicopathological parameters

The main results of the relationship between LMR and clinicopathological parameters are summarized in Table 3. A low LMR was associated with an advanced tumor progression, specifically with a high histological grade (G2/G3 vs. low G1 grade; OR: 1.67; 95% CI: 1.26–2.20; *p* < 0.001) and late FIGO stages (III-IV vs. early I-II stages; OR: 3.55; 95% CI: 2.68–4.70; p < 0.001), as well as with the presence of malignant ascites (OR: 1.87; 95% CI: 1.11–3.14; *p* = 0.02) and lymph node metastasis (OR: 1.70; 95% CI: 1.13–2.54; *p* = 0.01). Similarly, a low LMR was related to a high serum CA-125 marker (> median vs. < median; OR: 2.18; 95% CI: 1.71–2.77; p < 0.001). However, no obvious association was found between LMR and patient age (> median vs. < median; OR: 1.18; 95% CI: 0.97–1.44; *p* = 0.09), histological type (serous vs. others; OR: 1.07; 95% CI: 0.88–1.30; *p* = 0.51), and evidence of optimal debulking (OR: 1.13; 95% CI: 0.71–1.80; *p* = 0.62).

**Table 3:** Meta-analysis of the association between LMR and clinicopathological features of ovarian cancer

Characteristics	No. of studies	No. of patients	OR (95% CI)	p	Heterogeneity	
					I^2^ (%)	Ph
Age (≥ 60 vs. < 60)	3	626	2.07 (1.22–3.50)	0.007	42	0.18
Gender (male vs. female)	4	4818	1.18 (0.68–2.04)	0.56	70	0.02
Smoking status (ever/current vs. never)	2	394	0.95 (0.63–1.45)	0.82	0	0.80
Differentiation (low vs. moderate/high)	5	4886	1.60 (1.10–2.32)	0.01	35	0.19
Tumor size (> 3 cm vs. < 3 cm)	2	496	1.86 (0.74–4.71)	0.19	71	0.06
T stage (III-IV vs. I-II)	3	4390	1.13 (1.01–1.28)	0.04	0	0.79
Lymph node metastasis (yes vs. no)	3	4390	1.22 (1.06–1.39)	0.005	0	0.67
Distant metastasis (yes vs. no)	1	124	1.46 (0.37–5.73)	0.59	–	–
Multiplicity (multiple vs. solitary)	2	496	1.04 (0.68–1.58)	0.86	0	0.49
Concomitant Cis (yes vs. no)	2	4322	0.88 (0.78–0.99)	0.03	0	0.87

*Cis* carcinoma in situ

#### Sensitivity analysis

A sensitivity analysis was performed to evaluate the stability/robustness of the meta-analysis results. We found that none of the individual studies substantially altered the combined HRs of all studies, suggesting that the conclusions are relatively reliable.

## Discussion

The present study is, to our knowledge, the most comprehensive, up-to-date, and with the largest sample size meta-analysis undertaken to estimate the prognostic value of LMR in ovarian cancer. According to the pooled results confirmed by subgroup analysis, there was a significant association between low LMR and poor survival outcome, specifically poor OS and PFS, in ovarian cancer patients. Therefore, it can be concluded that LMR is an independent prognostic factor in ovarian cancer.

Additionally, in this study, the correlations between LMR and several clinicopathological parameters were evaluated. We found that a low LMR was highly correlated with tumor high G2/G3 histological grades and late III-IV FIGO stages, as well as with high serum CA-125 levels, and presence of malignant ascites and lymph node metastases, in agreement with the poor overall survival outcome associated with this ratio.

However, the potential mechanisms underlying the prognostic ability of LMR have not yet been clarified. It is known that lymphocytes play an important role in cell-mediated antitumor immune responses and in tumor immunological surveillance [26, 27]. Cytotoxic lymphocytes, mainly cytotoxic T cells, are crucial to eliminate residual cancer cells and, as such, are applied in immunotherapy [28, 29]. Monocytes seem to have an impact on tumorigenesis through differentiation into tumor-associated macrophages (TAMs). TAMs are major players in inflammation, being recruited to the tumor site in response to tumor-derived chemotactic factors [30]. Therefore, TAM levels may reflect the tumor burden. Moreover, recent studies reported that an increased local infiltration of TAMs is associated with a poor prognosis in several cancer types [31, 32]. In line with this, LMR may represent the balance between antitumor immune reaction and tumor promotion function. Thus, a low LMR would be associated with a favorable tumor progression, explaining at least in part our results.

Our study presents several limitations. First, all studies included were carried out in Asian countries, implying that more cohort studies from other regions are necessary. Second, our conclusions could have been influenced by the heterogeneity of the results of the studies included in this meta-analysis, as well as by unknown carcinogenesis mechanisms. Third, the cut-off value of LMR was not uniform across the studies analyzed. Finally, only retrospective studies were included, which might have introduced confounding variables; thus, control-test studies are missing. Nevertheless, the present meta-analysis, which is conceptually superior to individual investigations, included sufficient published studies with data from a large number of patients, allowing for adequate evaluation of the prognostic value of LMR in ovarian cancer.

## Conclusions

The present study revealed that a low pretreatment (baseline) LMR is associated with a poor OS and PFS in ovarian cancer patients, as well as with severe clinicopathological features including advanced tumor characteristics. Therefore, as LMR is easily accessible, it may be a useful prognostic biomarker in ovarian cancer and thus, relevant in the management of the disease.

## Data Availability

The datasets used and/or analyzed during the current study are available from the corresponding author on reasonable request.
